# Transferrin receptor-1 and ferritin heavy and light chains in astrocytic brain tumors: Expression and prognostic value

**DOI:** 10.1371/journal.pone.0182954

**Published:** 2017-08-24

**Authors:** Ann Mari Rosager, Mia D. Sørensen, Rikke H. Dahlrot, Steinbjørn Hansen, David L. Schonberg, Jeremy N. Rich, Justin D. Lathia, Bjarne W. Kristensen

**Affiliations:** 1 Department of Pathology, Odense University Hospital, Odense, Denmark; 2 Department of Clinical Research, University of Southern Denmark, Odense, Denmark; 3 Department of Oncology, Odense University Hospital, Odense, Denmark; 4 Department of Stem Cell Biology and Regenerative Medicine, Cleveland Clinic, Cleveland, United States of America; 5 Department of Cellular and Molecular Medicine, Cleveland Clinic, Cleveland, United States of America; University of Portsmouth, UNITED KINGDOM

## Abstract

Astrocytic brain tumors are the most frequent primary brain tumors. Treatment with radio- and chemotherapy has increased survival making prognostic biomarkers increasingly important. The aim of the present study was to investigate the expression and prognostic value of transferrin receptor-1 (TfR1) as well as ferritin heavy (FTH) and light (FTL) chain in astrocytic brain tumors. A cohort of 111 astrocytic brain tumors (grade II-IV) was stained immunohistochemically with antibodies against TfR1, FTH, and FTL and scored semi-quantitatively. Double-immunofluorescence stainings were established to determine the phenotype of cells expressing these markers. We found that TfR1, FTH, and FTL were expressed by tumor cells in all grades. TfR1 increased with grade (p<0.001), but was not associated with prognosis in the individual grades. FTH and FTL were expressed by tumor cells and cells with microglial/macrophage morphology. Neither FTH nor FTL increased with malignancy grade, but low FTH expression by both tumor cells (p = 0.03) and microglia/macrophages (p = 0.01) correlated with shorter survival in patients anaplastic astrocytoma. FTL-positive microglia/macrophages were frequent in glioblastomas, and high FTL levels correlated with shorter survival in the whole cohort (p = 0.01) and in patients with anaplastic astrocytoma (p = 0.02). Double-immunofluorescence showed that TfR1, FTH, and FTL were co-expressed to a limited extent with the stem cell-related marker CD133. FTH and FTL were also co-expressed by IBA-1-positive microglia/macrophages. In conclusion, TfR1 was highly expressed in glioblastomas and associated with shorter survival in the whole cohort, but not in the individual malignancy grades. Low levels of FTH-positive tumor cells and microglia/macrophages were associated with poor survival in anaplastic astrocytomas, while high amounts of FTL-positive microglia/macrophages had a negative prognostic value. The results suggest that regulation of the iron metabolism in astrocytic brain tumors is complex involving both autocrine and paracrine signaling.

## Introduction

Astrocytic brain tumors are the most frequent and aggressive brain tumors in adults [1]. The most common and aggressive type is the glioblastoma (GBM) having a median survival of 15 months [2, 3]. High-dose radiotherapy and temozolomide have increased survival [2, 3] making prognostic biomarkers in gliomas increasingly important. Tumor cells in GBM display a cellular hierarchy with brain tumor-initiating cells (BTIC) at the apex [4, 5]. BTIC are located in niches and thrive in stressful conditions such as hypoxia, inflammation, oxidative stress, and low supply of glucose [6–10]. Iron metabolism is thought to be involved in mechanisms supporting these conditions [11–13]. Deregulation of the iron homeostasis has also been linked to other cancers as well as neuro-degenerative diseases [14].

Iron is an important co-factor required for biological activity of many enzymes, and the iron metabolism is strictly controlled to avoid free iron toxicity [15]. Iron enters the brain across the blood-brain-barrier, and uptake in the capillary endothelial cells is regulated by transferrin receptor-1 (TfR1) [16]. A similar regulation has been reported in cancer cells [17]. Intracellular iron is stored by two subtypes of the protein ferritin that differ in their subunits, either ferritin heavy (FTH) or light (FTL) chain [18]. FTH is important for rapid uptake and reutilization of iron, while FTL is associated with long-term storage [19].

High expression of TfR1 has been observed in different cancers including breast, lung, and bladder cancer as well as in malignant gliomas [17]. In The Cancer Genome Atlas (TCGA) dataset, a negative correlation was found between overall survival and high mRNA levels of TfR1, FTL, and FTH in gliomas. Recently, similar results were obtained when the three iron-related proteins were investigated on tissue microarrays (TMAs) containing tissue from 95 gliomas of oligodendroglial and astrocytic subtype [20]. Supporting BTIC functions, TfR1-positive GBM cells formed tumorspheres more frequently than TfR1-negative cells *in vitro*. Moreover, *in vivo* xenografting showed that mice implanted with TfR1^high^ GBM cells developed tumors earlier than mice implanted with TfR1^low^ GBM cells [20]. BTICs sorted based on the expression of CD133 showed elevated levels of TfR1, FTH, and FTL compared to non-BTICs. When short hairpin RNA interference was used to ablate ferritin activity, decreased BTIC proliferation was observed. Further, implanting BTICs in mice containing FTH or FTL knockdown resulted in sparse or absent tumor formation [20].

The aim of the present study was to investigate the expression and prognostic value of TfR1, FTL, and FTH in a patient cohort of 111 astrocytomas. Immunohistochemical staining was performed on whole slides as expression of BTIC-related markers may be under- or overestimated in TMAs due to the heterogeneity of gliomas [21]. Also, a possible expression of FTL and FTH by microglia/macrophages could be taken more comprehensively into account. In addition, we wanted to investigate the prognostic potential of TfR1, FTL, and FTH in individual glioma grades, since this aspect is of high clinical interest. To determine the phenotype of TfR1-, FTL-, and FTH-positive cells, a panel of double-immunofluorescence stainings was established consisting of the BTIC-related markers CD133 [22, 23] and nestin [24–26], the astrocytic marker glial fibrillary acidic protein (GFAP), and the microglial/macrophage marker ionized calcium-binding adapter molecule 1 (IBA-1).

## Material and methods

### Odense University Hospital (OUH) astrocytoma cohort

The tumor tissue samples (n = 111) were obtained from patients diagnosed with primary astrocytoma and included 70 GBMs (World Health Organization (WHO) grade IV), 18 anaplastic astrocytomas (AAs) (WHO grade III), and 23 diffuse astrocytomas (DAs) (WHO grade II). All patients underwent initial surgery between 1994 and 2005 at OUH, Denmark. None of the patients received treatment prior to the surgery. To ensure a sufficient and representative amount of high quality viable tumor tissue, we excluded: tumor tissue removed by ultrasonic aspiration, gliomas where all the material had been frozen before paraffin embedding, and biopsies where the material measured less than 4 mm. The tumors were re-evaluated by two independent neuropathologists according to the WHO guidelines 2007 [27]. Patient characteristics are presented in **Table 1**.

**Table 1:** Clinicopathological characteristics of patient samples.

	All astrocytomas	DA	AA	GBM
**Patients (n)**	111	23	18	70
**OS (months)**				
Median	10.5	55.5	18.4	8.4
Range	(0.07–241.3)	(2.1–241.3)	(2.2–110.1)	(0.07–153.4)
**Age (years)**				
Median	58	45	57	61
Range	(2–78)	(2–75)	(14–77)	(21–78)
**Sex (male/female)**	70/41	15/8	11/7	44/26
**Endpoint (alive/dead)**	6/105	5/18	0/18	1/69

*Abbreviations*: AA anaplastic astrocytoma, DA diffuse astrocytoma, GBM glioblastoma, OS overall survival.

### Tissue

Fresh tissue biopsies were fixed in 4% neutral buffered formaldehyde and paraffin embedded. Three μm sections were cut on a microtome and stained routinely with haematoxylin-eosin to define representative tumor regions.

Normal brain tissue specimens for staining controls were obtained from two adult autopsies. Cause of death was not related to any CNS diseases. The tissue was fixed in 4% neutral buffered formaldehyde for 24 hours.

The official Danish ethical review board named the Regional Scientific Ethical Committee of the Region of Southern Denmark approved the use of human glioma tissue (permission J. No. S-2011 0022). Use of the tissue was not prohibited by any of the patients according to the Danish Tissue Application Register.

### Immunohistochemistry

Three μm paraffin sections were stained on a Dako Autostainer Universal Staining System (Dako, Glostrup, Denmark). The sections were deparaffinized, and heat induced epitope retrieval (HIER) was performed by incubation in a buffer solution consisting of 10 mmol/L Tris-base and 0.5 mmol/L EGTA, pH 9. After blocking of endogenous peroxidase activity by incubation in 1.5% hydrogen peroxide, the sections were incubated for 60 min with primary antibodies against TfR1 (CD71) (10F11, 1+50, NovoCastra, Newcastle, United Kingdom), FTH (ab65080, 1+800, Abcam, Cambridge, United Kingdom), or FTL (ab69090, 1+1000, Abcam). The antigen-antibody complex was detected using EnVision (Dako). Visualization was performed using DAB (diaminobenzidine) as chromogen. Finally, sections were counterstained with Mayer’s hematoxylin, and coverslips were mounted with Aquatex. Paraffin sections from a TMA containing 28 normal tissues and 12 cancers were used as controls. Omitting primary antibodies served as negative controls as well as controls for non-specific staining related to the detection system.

### Double-immunofluorescence

Double-immunofluorescence stainings were performed on a TMA containing tissue from nine GBMs, normal colon, placenta, cerebellum, and rat hippocampus as previously described [20, 28, 29]. The preparations as well as the first step in the stainings were as described above. After detection of TfR1 (CD71) (1+200), FTH (1+12000), or FTL (1+16000) using Catalyzed Signal Amplification II kit with FITC (CSA II, Dako), a second round of HIER followed by quenching of endogen peroxidase was performed. Sections were then incubated with antibodies against nestin (96908, 1+200, R&D systems, Minneapolis, Minnesota, USA), CD133 (W6B3C1, 1+40, Miltenyi Biotec, Teterow, Germany), GFAP (Z0334, 1+8000, Dako), or IBA-1 (019–19741 1+12000, Wako Pure Chemical Industries, Osaka, Japan). Tyramide Amplification Signal Cyanine 5 (TSA-Cy5, Perkin Elmer, Waltham, Massachusetts, USA) was used as detection system. Sections were mounted with VECTASHIELD Antifade Mounting Medium with 4.6-diamidino-2-phenylindole (DAPI, VWR International, Radnor, Pennsylvania, USA). Fluorescent images were taken with a Leica DM6000B microscope connected to an Olympus DP72 1.4 Mega Pixel CCD (Olympus, Tokyo, Japan) camera using DAPI (Omega XF06, Omega Optical, Brattleboro, Vermont, USA), FITC (Leica, Wetzlar, Germany) and Cy5 (Omega XF110-2) filters.

### Pathological scoring

The immunohistochemical stainings were assessed using semi-quantitative scoring on whole slides. Prior to scoring of the whole cohort, ten tumors were evaluated and scored repeatedly to ensure reproducibility. Before the scoring was performed, a set of criteria was established and carefully followed (**Table 2**). For each individual staining, two parameters, “tumor cell fraction” and “tumor cell intensity”, were assessed and given a score from 0–2 with 0 indicating negative staining, 1 moderate staining, and 2 strong staining. The parameter “tumor cell score” was defined as a sum of the tumor cell fraction score and tumor cell intensity score with a maximum score of 4. In the ferritin stainings, positive cells with microglial/macrophage morphology were observed, and two additional parameters were therefore scored: “microglial fraction” and “microglial intensity”. The parameter “microglial score” was defined as a sum of the microglial fraction score and microglial intensity score with a maximum score of 4. Cells with microglial/macrophage morphology were not incorporated into the tumor cell scores. Necrotic areas and infiltration zones were not included in the assessment. Patient data are available in **S1 File**.

**Table 2:** Pathological scoring of TfR1, FTH, and FTL.

**TfR1 score**	**0**	**1**	**2**
Positive tumor cell fraction	0% to <2%	2% to < 75%	75% to 100%
Tumor cell intensity	None	Faint	Intense
**FTH score**	**0**	**1**	**2**
Positive tumor cell fraction	0% to <2%	2% to < 75%	75% to 100%
Tumor cell intensity	None	Faint	Intense
Positive microglial fraction	0% to <2%	2% to < 75%	75% to 100%
Microglial intensity	None	Faint	Intense
**FTL score**	**0**	**1**	**2**
Positive tumor cell fraction	0% to <2%	2% to < 75%	75% to 100%
Tumor cell intensity	None	Faint	Intense
Positive microglial fraction	0% to <2%	2% to < 75%	75% to 100%
Microglial intensity	None	Faint	Intense

For each tumor, a tumor cell score for TfR1, FTH, and FTL was calculated by adding up the two tumor scores: positive tumor cell fraction and tumor cell intensity, resulting in a maximum score of 4. Similarly, a microglial score for FTH and FTL was calculated by adding up the two microglial scores: positive microglial fraction and microglial intensity resulting in a maximum score of 4.

*Abbreviations*: FTH ferritin heavy chain, FTL ferritin light chain, TfR1 transferrin receptor-1.

### *In silico* analyses of the Cancer Genome Atlas (TCGA)

TCGA gene expression datasets for TfR1 (TFRC), FTH (FTH1), FTL, and IBA-1 (AIF-1) were exported from GlioVis (https://gliovis.bioinfo.cnio.es). Data were available for 497 patients with primary GBM [30]. Datasets used in the present study are available in **S2 File**.

### Statistical analysis

Tumor cell score and microglial score were compared with tumor grades using the non-parametric Kruskal Wallis test followed by Dunn's multiple comparisons test. Overall survival was defined as time from primary surgery until death or date of censoring (April 10^th^, 2015). The univariate relationships were illustrated by Kaplan-Meier plots, and differences in overall survival were analyzed using Log Rank tests. This was performed for all WHO grades combined and for DAs, AAs, and GBMs separately. Multivariate Cox proportional hazard regression analyses including age and sex were performed for GBMs. For survival analyses, patients were grouped into two, based on the summed scores with score 0–2 representing the low expression group and score 3–4 representing the high expression group. All assumptions were tested, and all analyses were carried out in STATA (StataCorp LP, Bryan, Texas, USA).

Correlations between IBA-1 gene expression, GBM subtypes, and expression of the three iron-related genes were investigated using the non-parametric Spearman’s correlation. Using the TfR1, FTH, and FTL datasets, survival analyses were carried out for each GBM subtype with the median as cutoff value.

## Results

### Staining in normal brain

**Fig 1:**
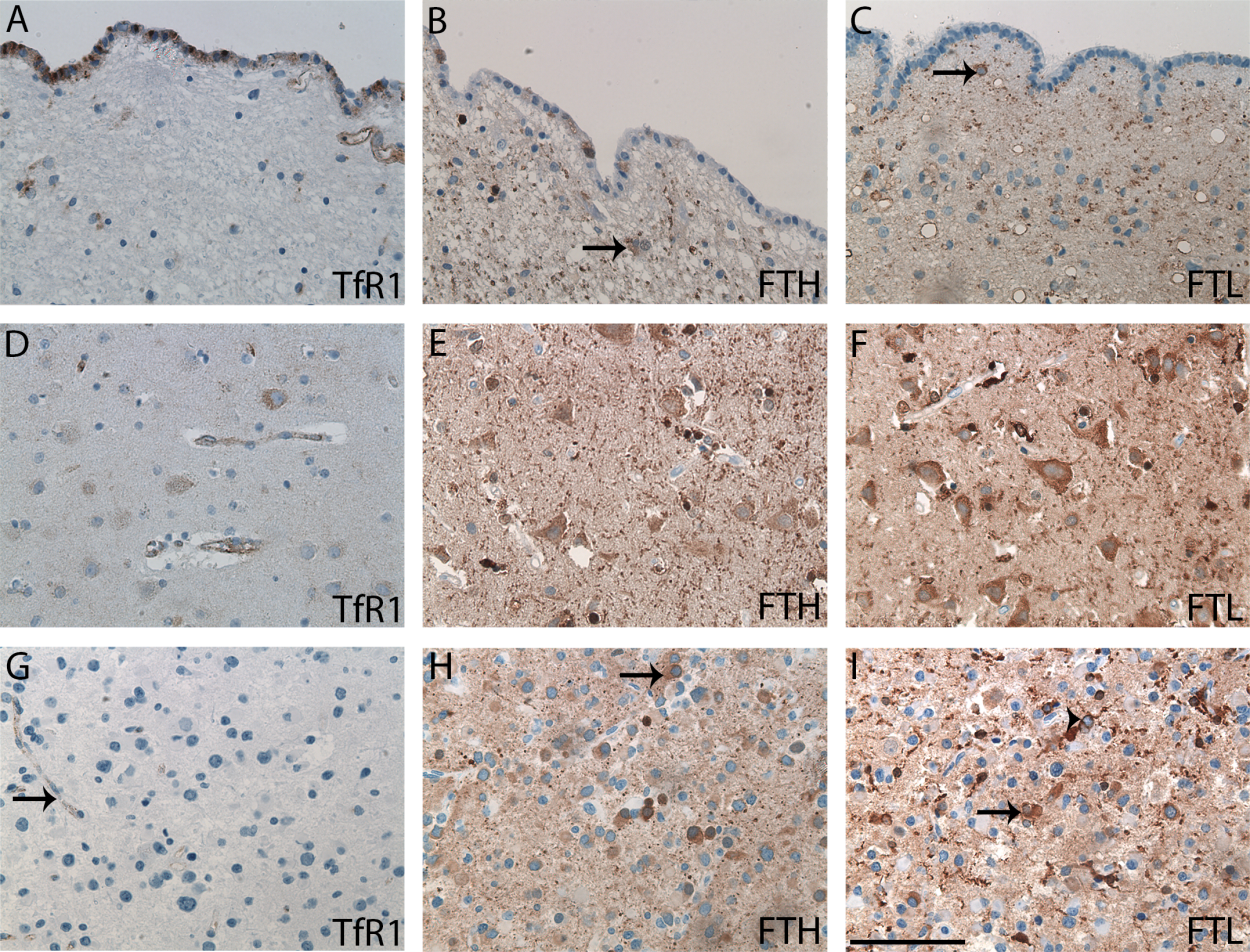
Normal brain tissue from subventricular zone and neocortex as well as DAs immunohistochemically stained with TfR1, FTH, and FTL. Cells in the ependymal layer expressed TfR1 **(A)** and FTH (**B**). No expression of FTL **(C)** in the ependymal layer was seen. A few cells beneath the ependymal layer were positive for FTH **(B)** and FTL **(C)** (arrows). In the neocortex, there was a weak TfR1 neuronal staining **(D)**, but a stronger neuronal staining for FTH **(E)** and FTL **(F)**, which also stained the neuropil. In DAs, TfR1was mainly expressed by the endothelium (arrow) **(G)**. The FTH **(H)** and FTL **(I)** stainings were pronounced in many DAs with staining of both tumor cells (arrows) and microglia (arrowheads). *Abbreviations*: DA diffuse astrocytoma, FTH ferritin heavy chain, FTL ferritin light chain, TfR1 transferrin receptor-1. Scale bar: 100 μm.

In the subventricular zone TfR1-positive cells were observed in the ependymal layer (**Fig 1A**), while few cells in and beneath the ependymal layer were FTH-positive (**Fig 1B**). The ependymal cells did not express FTL, but a few cells beneath the ependymal expressed FTL (**Fig 1C**). For TfR1, neurons in the neocortex had a weak expression (**Fig 1D**), and endothelium was mainly positive stained in the small capillaries. For FTH, neurons in neocortex showed a moderate expression, and the neuropil had a weak staining, while some cells morphologically resembling microglia had a stronger staining (**Fig 1E**). A similar staining pattern was found for FTL (**Fig 1F**).

### Tumor cell staining in astrocytomas

**Fig 2:**
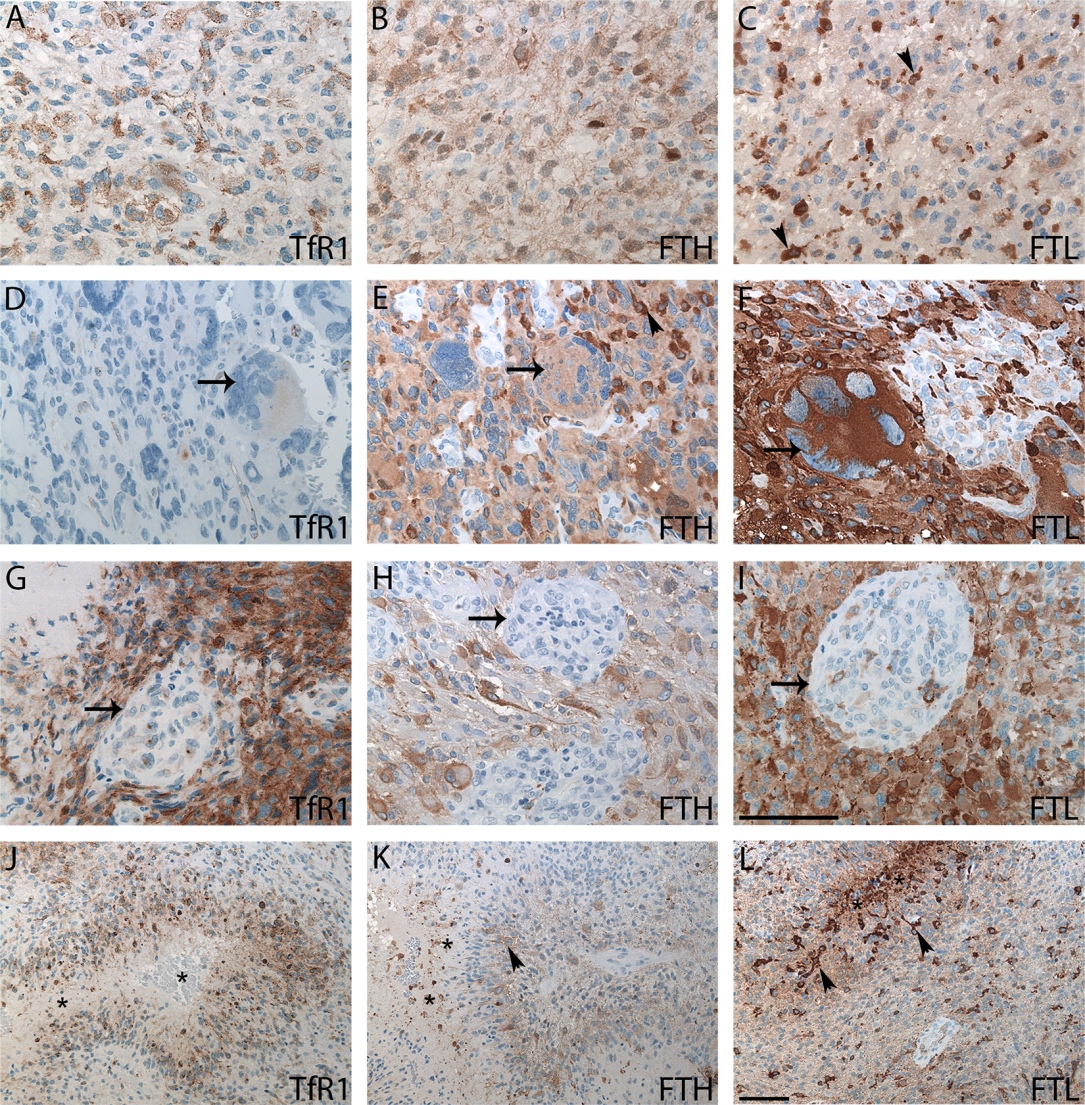
AAs and GBMs immunohistochemically stained with TfR1, FTH, and FTL. AAs showed weak TfR1staining **(A)** and stronger FTH **(B)** and FTL **(C)** staining. GBMs with giant cells were weakly positive for TfR1 **(D)**, moderately positive for FTH **(E),** and strongly positive for FTL **(F)**. GBMs had no or limited glomeruloid staining for TfR1 **(G)**, FTH **(H)**, or FTL **(I)** (arrows). In GBMs with pseudo-palisading necroses (asterisks) TfR1-positive **(J)**, FTH-positive **(K)**, and FTL-positive **(L)** cells were observed in perinecrotic areas. Cells with microglial/macrophage morphology were easily identified in both the FTH (arrowheads) (**E** and **K**) and FTL stainings (arrowheads) (**C** and **L**). *Abbreviations*: AA anaplastic astrocytoma, FTH ferritin heavy chain, FTL ferritin light chain, GBM glioblastoma, TfR1 transferrin receptor-1. Scale bar: (A-I) 100 μm, (J-L) 100 μm.

In most DAs, TfR1 was not present (**Fig 1G**), while FTH and FTL expression patterns were heterogeneous with strong, diffuse expression in some tumors and weak expression in others (**Fig 1H** and **Fig 1I**). Likewise most AAs were TfR1-negative, but some expressed the protein (**Fig 2A**), and FTH and FTL was heterogeneously expressed (**Fig 2B** and **Fig 2C**). GBM cells were positive for TfR1 with a heterogeneous expression pattern containing regions of high intensity with multiple positive tumor cells as well as regions without any TfR1-expressing cells. Multinucleated giant cells showed a weak TfR1 expression (**Fig 2D**), but had an intense expression of FTH and especially FTL (**Fig 2E** and **Fig 2F**).

TfR1-positive tumor cells were often seen near blood vessels (**Fig 2G**), and in all tumors, the endothelium of small blood vessels expressed TfR1 thereby serving as an internal positive control. In contrast, blood vessels including glomeruloid vessels lacked expression of FTH (**Fig 2H**) and FTL (**Fig 2I**). Cells surrounding pseudo-palisading necrosis expressed TfR1 (**Fig 2J**), FTH (**Fig 2K**) as well as FTL (**Fig 2L**).

In general, the expression patterns of FTH and FTL appeared very similar, although more cells were positive for FTL, and the intensity of the positive cells was often stronger than that of FTH-positive cells. The cells with the strongest FTL and FTH intensities resembled microglia/macrophages exhibiting microstellate or macrophage morphology.

### Association with grade and prognostic impact of tumor cell score in astrocytomas

**Fig 3:**
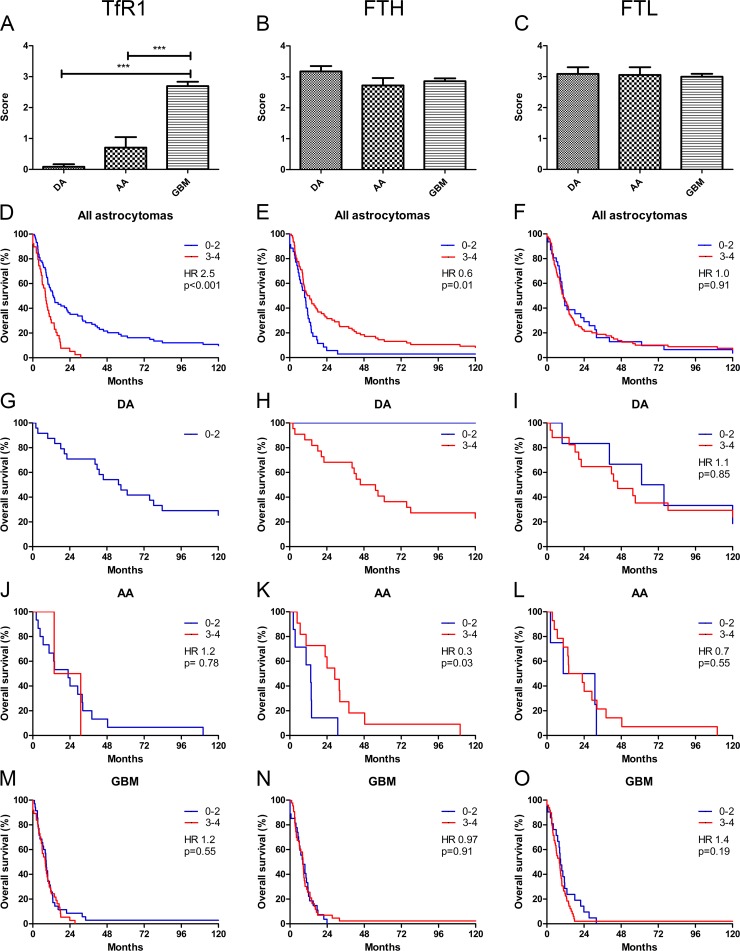
Association of TfR1, FTH, and FTL tumor cell score with grade and overall survival. TfR1 tumor cell score was found to increase with grade **(A)**. No increase was observed for FTH **(B)** and FTL **(C)**. Combining all astrocytomas, high expression of TfR1 was associated with poor overall survival **(D)**, and low expression of FTH was associated with poor overall survival **(E)**. No association was seen for FTL **(F)**. In the TfR1 staining, no DAs had a tumor cell score greater than 2 explaining the presence of only one survival curve **(G)**. In the FTH staining, only one tumor had low tumor cell score **(H)**. No association with overall survival was observed for FTL in DAs **(I)**. In AAs, no association with overall survival was observed for TfR1 **(J)** or FTL **(L)**, while low expression of FTH was associated with poor overall survival **(K)**. In GBMs, no significant association with overall survival was observed for TfR1 **(M)**, FTH **(N)**, or FTL **(O)**. Tumor scores are shown with error bars representing SEM. *** corresponds to p<0.001. *Abbreviations*: AA anaplastic astrocytoma, DA diffuse astrocytoma, FTH ferritin heavy chain, FTL ferritin light chain, GBM glioblastoma, TfR1 transferrin receptor-1.

The TfR1 tumor cell score increased with malignancy grade (p<0.001) (**Fig 3A**). Neither FTH (**Fig 3B**) nor FTL (**Fig 3C**) increased with grade.

When combining all grades, high TfR1 tumor cell score was associated with a poor prognosis (HR 2.5; 95% CI 1.59–3.79; p<0.001) (score 0–2: n = 74 and score 3–4: n = 39) (**Fig 3D**). Differences in overall survival based on TfR1 expression could not be compared in DAs, since all DAs received a low tumor cell score (**Fig 3G**). TfR1 tumor cell score was not associated with overall survival in AAs (HR 1.2; 95% CI 0.27–5.69; p = 0.78) (score 0–2: n = 15 and score 3–4: n = 2) (**Fig 3J**) or in GBMs (HR 1.2; 95% CI 0.72–1.86; p = 0.55) (score 0–2: n = 35 and score 3–4: n = 37) (**Fig 3M**). Multivariate analysis did not provide any additional information for GBMs (**Table 3**).

**Table 3:** Multivariate analysis for patients with GBMs (n = 70) with TfR1, FTH, and FTL tumor cell score.

	TfR1		FTH		FTL	
Variable	HR (95% CI)	p-value	HR (95% CI)	p-value	HR (95% CI)	p-value
**Age**	1.0 (0.99–1.04)	0.39	1.0 (0.98–1.03)	0.60	1.0 (0.98–1.03)	0.62
**Gender**						
Female	1.0		1.0		1.0	
Male	1.1 (0.66–1.82)	0.72	1.1 (0.66–1.82)	0.83	1.8 (1.09–2.99)	0.02
**TCS**						
0–2	1.0		1.0		1.0	
3–4	1.1 (0.70–1.84)	0.62	0.9 (0.54–1.59)	0.78	1.5 (0.90–2.66)	0.12

*Abbreviations*: CI confidence interval, FTH ferritin heavy chain, FTL ferritin light chain, HR hazard ratio, TCS tumor cell score, TfR1 transferrin receptor-1.

Combining all astrocytomas, high FTH tumor cell score was associated with better outcome (HR 0.6; 95% CI 0.37–0.87; p = 0.01) (score 0–2: n = 35 and score 3–4: n = 76) (**Fig 3E**). Only one DA had a low FTH tumor cell score preventing statistical comparison (**Fig 3H**). In AAs, a high FTH tumor cell score was associated with better outcome (HR 0.3; 95% CI 0.09–0.90; p = 0.03) (score 0–2: n = 7 and score 3–4: n = 11) (**Fig 3K**). No association with overall survival was observed in GBMs neither in univariate (HR 0.97; 95% CI: 0.60–1.59; p = 0.91) (score 0–2: n = 35 and score 3–4: n = 37) (**Fig 3N**) nor in multivariate analysis (**Table 3**).

FTL tumor cell score was not associated with overall survival in all astrocytomas combined (HR 1.0; 95% CI 0.67–1.56; p = 0.91) (score 0–2: n = 31 and score 3–4: n = 81) (**Fig 3F**), in DAs (HR 1.1; 95% CI 0.39–3.12; p = 0.85) (score 0–2: n = 6 and score 3–4: n = 17) (**Fig 3I**), or in AAs (HR 0.7; 95% CI 0.22–2.25; p = 0.55) (score 0–2: n = 4 and score 3–4: n = 14) (**Fig 3L**). In GBMs, FTL did not correlate with survival, however, the subgroup of patients with low FTL tumor cell score tended to have a better prognosis (HR 1.4; 95% CI 0.84–2.43; p = 0.19) (score 0–2: n = 21 and score 3–4: n = 49) (**Fig 3O**); but FTL did not have an independent prognostic value in GBMs (**Table 3**).

### Association with grade and prognostic impact of microglial score in astrocytomas

**Fig 4:**
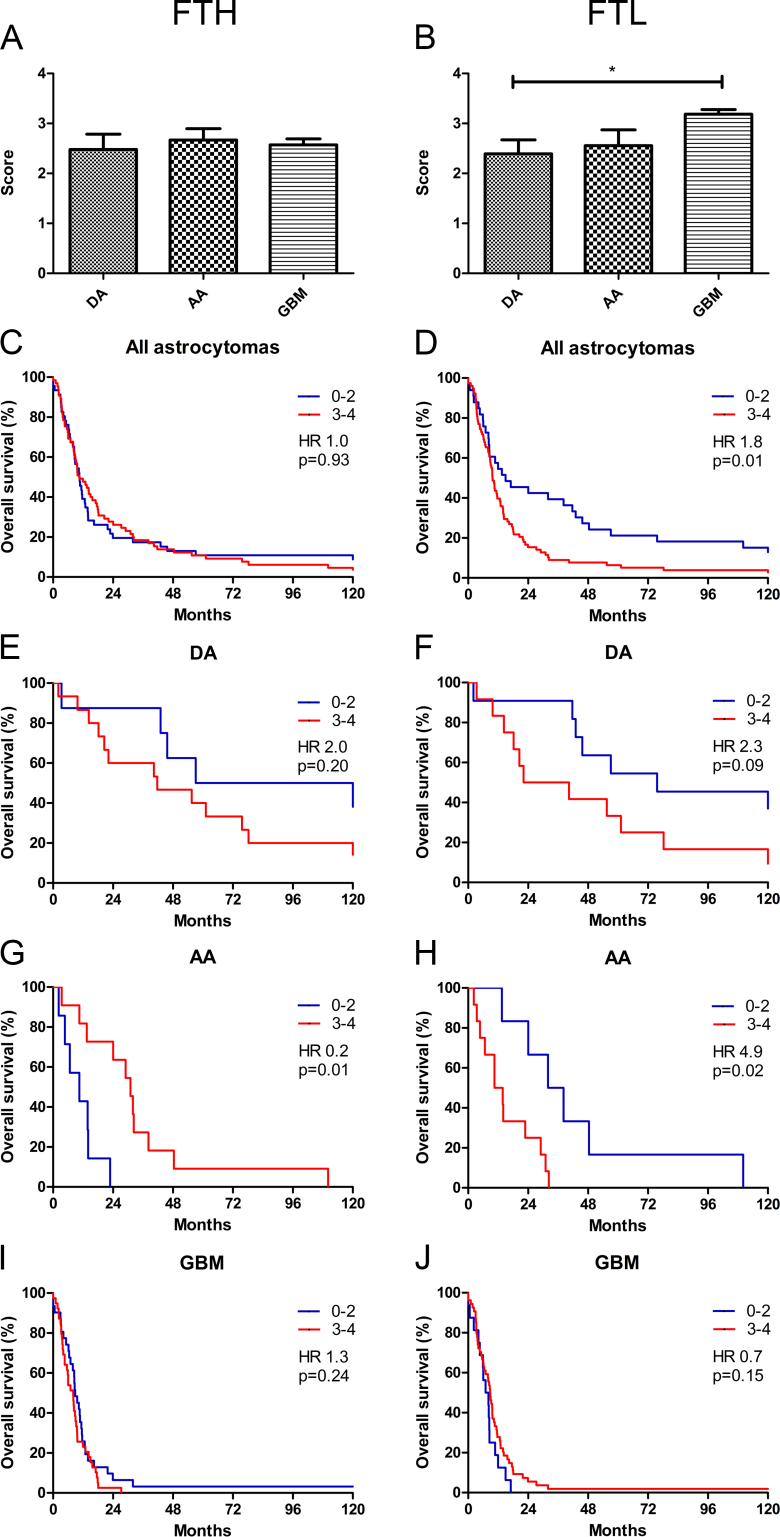
Association of FTH and FTL microglial score with grade and overall survival. FTH microglial score was similar across tumor grades **(A)**, whereas FTL microglial score increased with WHO grade **(B)**. Combining all astrocytomas, no association with survival was observed for FTH **(C)**, but high FTL microglial score was associated with poor survival **(D)**. No association with overall survival was seen for FTH **(E)** or FTL **(F)** in DAs. In AAs, high FTH was associated with longer overall survival **(G)**, whereas low FTL microglial score was associated with longer overall survival **(H)**. In GBMs, no association with overall survival was observed for FTH **(I)** or FTL **(J)**. Microglial scores are shown with error bars representing SEM. * corresponds to p<0.05. *Abbreviations*: AA anaplastic astrocytoma, DA diffuse astrocytoma, FTH ferritin heavy chain, FTL ferritin light chain, GBM glioblastoma.

The microglial score for FTH did not differ among the different grades (**Fig 4A**), whereas the FTL microglial score was significantly higher in GBMs as compared to DAs and AAs (p<0.05) (**Fig 4B**).

In DAs, no significant association between overall survival and FTH was observed (HR 2.0; 95% CI 0.70–5.57; p = 0.20) (score 0–2: n = 8 and score 3–4: n = 15) (**Fig 4E**). In AAs, high microglial score was associated with a better overall survival (HR 0.2; 95% CI 0.04–0.64; p = 0.01) (score 0–2: n = 7 and score 3–4: n = 11) (**Fig 4G**), while no association with overall survival was observed for patients with GBM in either in the univariate (HR 1.3; 95% CI 0.82–2.18; p = 0.24) (score 0–2: n = 31 and score 3–4: n = 39) (**Fig 4I**) or multivariate analyses (**Table 4**).

**Table 4:** Multivariate analysis for patients with GBMs (n = 70) with FTH and FTL microglial score.

	FTH		FTL	
Variable	HR (95% CI)	p-value	HR (95% CI)	p-value
**Age**	1.0 (0.80–2.18)	0.78	1.0 (0.98–1.03)	0.65
**Gender**				
Female	1.0		1.0	
Male	1.0 (0.61–1.68)	0.96	1.8 (1.09–2.99)	0.05
**MS**				
0–2	1.0		1.0	
3–4	1.3 (0.80–2.18)	0.27	0.7 (0.40–1.28)	0.26

*Abbreviations*: CI confidence interval, FTH ferritin heavy chain, FTL ferritin light chain, HR hazard ratio, MS microglial score

Low FTL microglial score was associated with a better overall survival in all astrocytomas combined (HR 1.8; 95% CI 1.56–2.79; p = 0.01) (**Fig 4D**) (score 0–2: n = 33 and score 3–4: n = 79), and a similar trend was observed in DAs (HR 2.3; 95% CI 0.88–6.81; p = 0.09) (score 0–2: n = 11 and score 3–4: n = 12) (**Fig 4F**). In AAs, low FTL microglial score was associated with better outcome (HR 4.9; 95% CI 1.31–17.95; p = 0.02) (**Fig 4H**) (score 0–2: n = 6 and score 3–4: n = 12), while no association was found in GBMs (HR 0.7; 95% CI 0.37–1.16; p = 0.15) (score 0–2: n = 16 and score 3–4: n = 54) (**Fig 4J**). Multivariate analysis of GBMs did not provide any additional information (**Table 4**).

### Double-immunofluorescence

**Fig 5:**
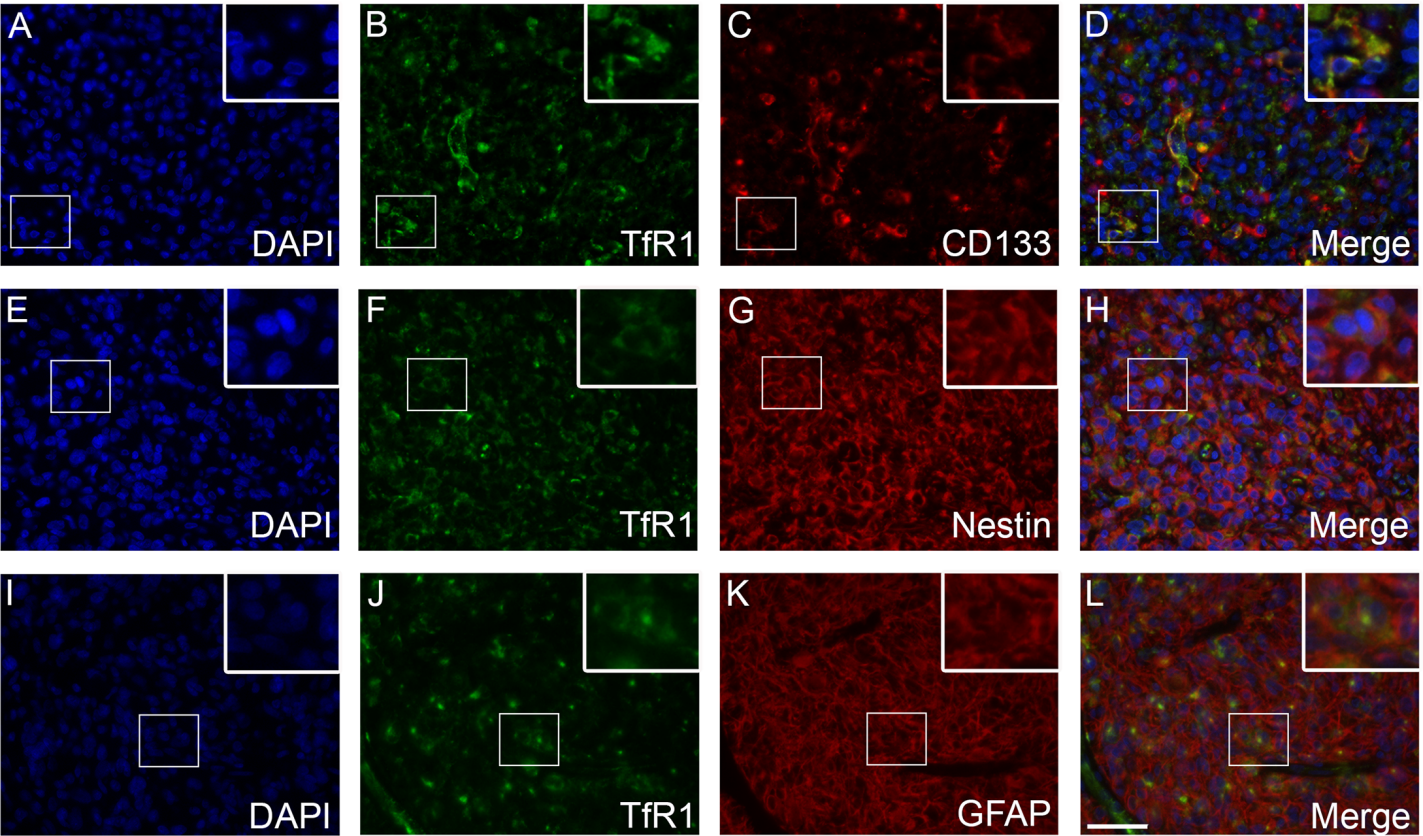
TfR1 double-immunofluorescence stainings. Co-expression of TfR1 and CD133 was observed in a number of tumor cells **(A-D)**. Some tumor cells showed limited co-expression with nestin **(E-H)**. A few tumor cells expressed both TfR1 and GFAP **(I-L)**. *Abbreviations*: GFAP glial fibrillary acidic protein, TfR1 transferrin receptor-1. Scale bar: 50 μm.

**Fig 6:**
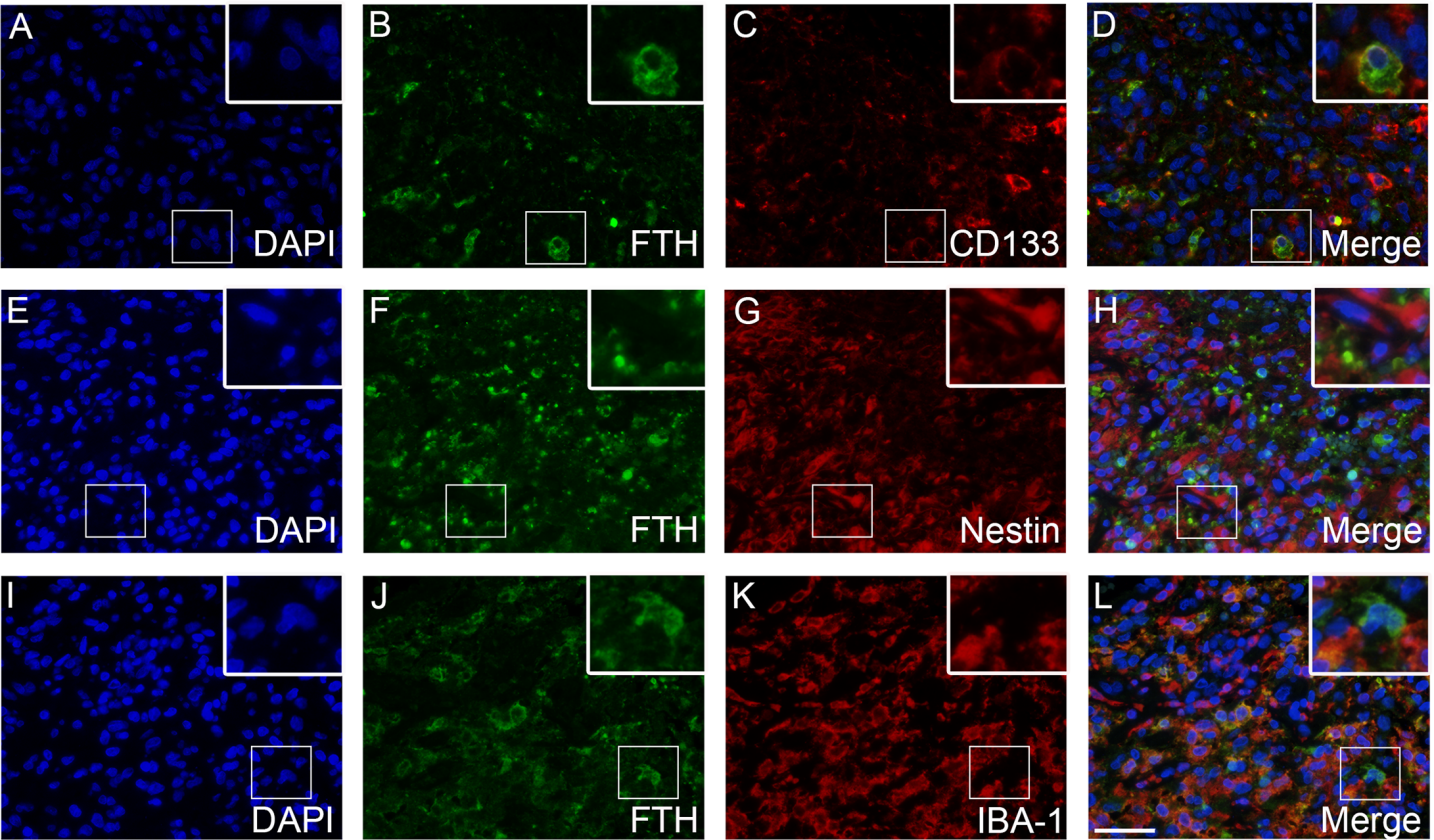
FTH double-immunofluorescence stainings. Co-expression of FTH and CD133 was observed in a number of tumor cells **(A-D)**. Co-expression of FTH and nestin was not observed **(E-H)**. Co-expression was often seen for FTH and the microglial/macrophage marker IBA-1 **(I-L)**. *Abbreviations*: FTH ferritin heavy chain, IBA-1 ionized calcium-binding adapter molecule 1. Scale bar 50 μm.

**Fig 7:**
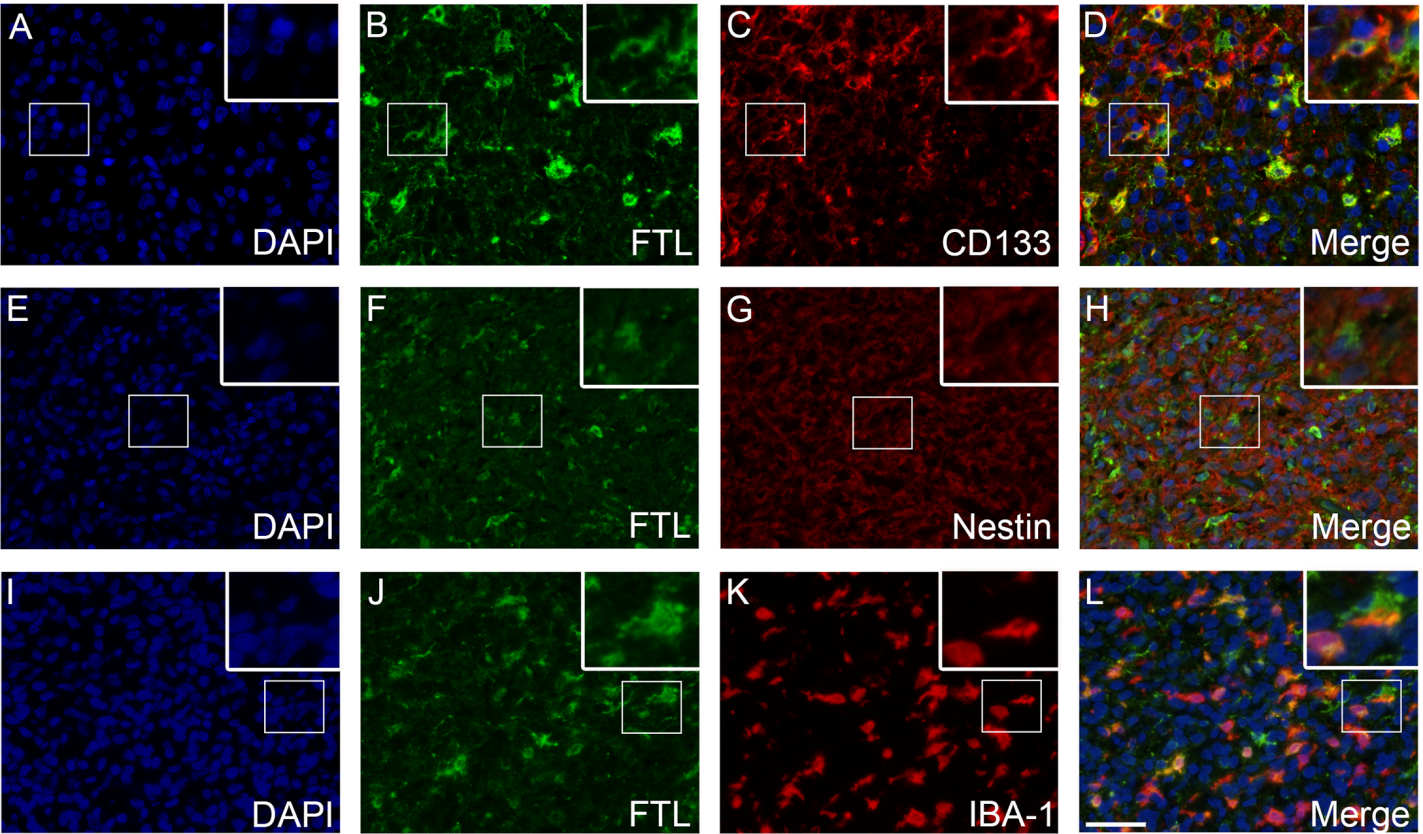
FTL double-immunofluorescence stainings. Co-expression of FTL and CD133 was observed in a number of tumor cells **(A-D)**. Co-expression of FTL and nestin was not observed **(E-H)**. Co-expression was often seen for FTL and the microglial/macrophage marker IBA-1 **(I-L)**. *Abbreviations*: FTL ferritin light chain, IBA-1 ionized calcium-binding adapter molecule 1. Scale bar 50 μm.

TfR1 was expressed by CD133-positive tumor cells (**Fig 5A–5D**) and to a limited degree by nestin-positive tumor cells (**Fig 5E–5H**). Tumor cells that co-expressed TfR1 and GFAP were also identified (**Fig 5I–5L**). The two ferritin stainings showed similar co-expression patterns. Few FTH-positive tumor cells co-expressed CD133 (**Fig 6A–6D**), while no co-expression between FTH and nestin was observed (**Fig 6E–6H**). Some IBA-1-positive cells co-expressed FTH (**Fig 6I–6L**). A similar co-expression pattern was observed for FTL (**Fig 7A–7L**).

### *In silico* analyses

**Fig 8:**
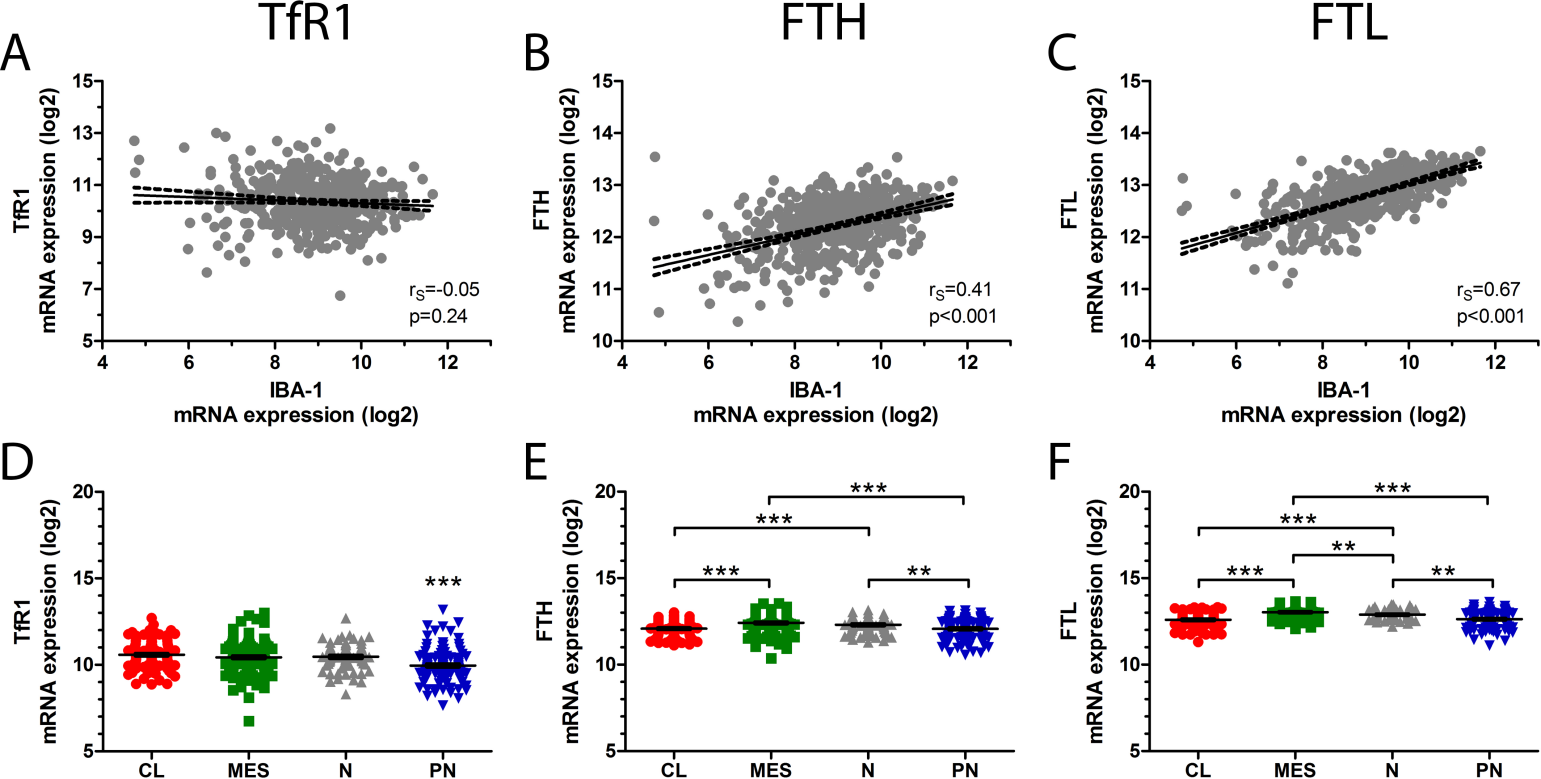
*In silico* analyses. IBA-1 mRNA expression did not correlate with TfR1 mRNA expression **(A)**, but showed positive correlation with both FTH **(B)** and FTL **(C)** mRNA expression levels. TfR1 expression was lowest in proneural GBMs **(D)**, while FTH **(E)** and FTL **(F)** levels were highest in mesenchymal and neural GBMs. Error bars represent SEM. * corresponds to p<0.05, ** p<0.01, and *** corresponds to p<0.001. *Abbreviations*: CL classical, FTH ferritin heavy chain, FTL ferritin light chain, GBM glioblastoma, IBA-1 ionized calcium-binding adapter molecule 1, MES mesenchymal, N neural, PN proneural, TfR1 transferrin receptor-1.

To investigate the relation between the iron-related molecules and microglia/macrophages further, correlation analyses between IBA-1 and TfR1, FTH, and FTL were performed using the TCGA dataset. No significant correlation was found between IBA-1 and TfR1 mRNA expression (r_s_ = -0.05; 95% CI 0.14–0.04; p = 0.24) (**Fig 8A**), while IBA-1 was positively correlated with both FTH (r_s_ = 0.41; 95% CI 0.34–0.49; p<0.001) (**Fig 8B**) and FTL (r_s_ = 0.67; 95% CI 0.61–0.71; p<0.001) (**Fig 8C**).

Investigating the mRNA expression levels of TfR1, FTH, and FTL in the four different GBM subtypes, TfR1 levels were found to be significantly lowest in the proneural subtype compared to the classical, mesenchymal, and neural subtypes (p<0.001) (**Fig 8D**). FTH expression was significantly higher in the mesenchymal and neural subtype compared to the classical (p<0.001) and proneural subtypes (p<0.001 and p<0.01, respectively) (**Fig 8E**), while FTL expression levels were highest in the mesenchymal subtype (p<0.001 or p<0.01) (**Fig 8F**).

Investigating the association between iron-related mRNA levels and survival in the different GBM subtypes showed that TfR1, FTH, and FTL had limited prognostic value in classical GBMs (p>0.05) (**S1A–S1C Fig**). In mesenchymal GBMs, only high levels of TfR1 negatively correlated with overall survival (HR 1.4; 95% CI 1.00–2.01; p = 0.049) (**S1D–S1F Fig**). In neural GBMs, overall survival was not associated with TfR1, FTH, or FTL (p>0.05) (**S1G–S1I Fig**). In proneural GBMs, high levels of TfR1 were significantly associated with shorter overall survival (HR 2.3; 95% CI 1.49–3.39; p<0.001) (**S1J Fig**); while FTH and FTL did not correlate with survival (p>0.05) (**S1K and S1L Fig**).

## Discussion

In our cohort, TfR1 expression was predominantly seen in GBMs, and high TfR1 expression was associated with worse prognosis analyzing all astrocytic brain tumors together. High TfR1 expression has also been observed in other cancers [31–34]. Recently, analysis of brain tumor data sets from Oncomine suggested that high mRNA levels of TfR1 were associated with high tumor grade. Analyzing the TCGA and the National Cancer Institute REpository for Molecular BRAin Neoplastic Data (NCI REMBRANDT) datasets, high TfR1 levels were found correlate with shorter overall survival in patients with glioma, and similar results were found when investigating protein expression data from grade II-IV TMAs [20]. In the present study, we performed a more comprehensive analysis and found no association between TfR1 expression and survival in the individual astrocytoma grades. This suggests that the prognostic significance of the iron-related proteins previously observed in all astrocytomas is related to WHO grade and does not provide additional prognostic information. We found that cells with tumor morphology co-expressed TfR1 and GFAP; additionally we observed co-expression of TfR1 and the stem cell-related markers CD133 and nestin, which is consistent with recently reported data demonstrating that GBM cells co-express TfR1 and the stem cell-related marker SOX2 [20]. Overall, these findings suggest that TfR1 may play a role at different levels of the tumor cell differentiation hierarchy; however, the association between TfR1 and cellular differentiation needs further investigation.

Almost all astrocytic tumors in our cohort expressed the two ferritin proteins. High FTH tumor cell expression was associated with a better prognosis in all astrocytomas, and this was also seen in AAs. In contrast, FTL was not associated with survival, and for both FTH and FTL no difference was observed among malignancy grades. These results are different from previous results obtained using TMAs composed of 95 grade II-IV gliomas of oligodendroglial and astroglial type, where high immunohistochemical scores for FTH and FTL were associated with shorter overall survival [20]. The discrepancy could be explained by inclusion of grade II and III oligodendroglial tumors in the TMA cohort, which is in contrast to our cohort containing only astrocytic tumors thereby reducing the risk of bias in the survival analysis. Focusing on GBMs and analyzing gene expression data from the TCGA dataset, high levels of FTH1 mRNA and FTL mRNA were previously found to be associated with poor outcome [20]. However, apparently these findings do not extend to the protein level as no association with survival was found in our data or in the TMA study [20]. In breast cancer, both high levels of FTH and FTL have been suggested to be associated with poor outcome [35]. However, in triple-negative breast cancer, high FTH expression was a favorable prognostic factor if the protein was present in the cytoplasm [36], thereby adding to the complexity of understanding and using iron-related proteins as biomarkers. In the context of gliomas, the four different molecular GBM subtypes may influence survival analysis in GBMs, especially when working with smaller patient cohorts [37]. To elucidate whether the expression levels of TfR1, FTH, and FTL differed among the GBM subtypes, we used the TCGA dataset and found that neural and especially mesenchymal GBMs overall had the highest expression levels of the three iron-related genes. When investigating the association between the three iron-related genes and overall survival in the four different GBM subtypes, high mRNA levels of TfR1 significantly correlated with overall survival, but only in the mesenchymal and proneural subtypes. Thus, accounting for GBM subtype in the survival analysis performed on our patient cohort could increase the prognostic significance of the three iron-related proteins, especially TfR1, although a larger cohort would be needed to ensure statistical significance.

More recently, a study from Wu et al. showed that FTL was elevated in GBM and silencing FTL *in vitro* inhibited the cell proliferation [38]. Gautam et al. found that the plasma levels of FTL were higher in GBM patients compared to healthy controls; however, this could also reflect that FTL is a marker for inflammation [39]. Altogether, these studies suggest that FTL plays a role in glioma biology, but the precise role of FTL needs to be further investigated.

FTH and FTL were expressed by a few CD133-positive cells suggesting that ferritins may not play a crucial role in the BTIC phenotype. However, in a recent study ferritins were found to be co-expressed to some extent with SOX2 [20]. In contrast, we did not observe any co-expression between the ferritins and nestin. This could be due the hierarchy that exists within the BTIC population as nestin may be expressed primarily by cells committed to the neural lineage, while CD133 and especially SOX2 may be expressed by more immature cells [40]. However, this possible relation between BTIC and iron-related proteins needs further investigation. Interestingly, we also found co-expression between ferritins and IBA-1, a specific marker of microglia and macrophages suggesting an important role of microglia/macrophages in brain tumor iron homeostasis which has also been reported by other groups [41–43]. Reportedly, microglia/macrophages are more prevalent in the mesenchymal subtype of GBM [44] suggesting that the microglial/macrophage contribution to the iron homeostasis is most significant in this GBM subtype. In return, iron status was found to influence microglial release of pro-inflammatory cytokines [45]. We approached the potential microglial contribution to glioma survival by scoring cells with a morphology resembling microglia/macrophages in the two immunohistochemical ferritin stainings. We observed that a high microglial score in the FTL staining was associated with poor prognosis in all grades combined as well as in AAs. In contrast, low FTH microglial score was associated with poor outcome in AAs. This observation could be explained by microglial phenotype e.g. polarization of microglia towards a pro-inflammatory or an anti-inflammatory phenotype thereby potentially influencing survival. Accordingly, iron-related proteins may play different roles in different cell types highlighting the complexity of interpreting expression of iron-related proteins and using these proteins as biomarkers.

Using iron-related proteins as targets may also be challenging. Several drugs targeting TfRs have been developed and tested in different cancers. In gliomas, phase I and II clinical trials with the TfR ligand transferrin conjugated to diphtheria toxin have been conducted on patients with recurrent GBMs showing promising results [46, 47]. However, in a phase III trial the drug failed mainly due to CNS toxicity [48, 49]. Systematic interrogation of iron flux recently showed that tumor stem cells require TfR1 and ferritins to propagate and form tumors in vivo [20] supporting that strategies compromising iron flux could be successful since the aspect of the tumor stem cells is taken into account. Using whole slides of non-cultured patient tumor tissue, our results supported this showing that TfR1, FTH, and FTL were co-expressed to some extent with the stem cell marker CD133, and we furthermore found that TfR1 was elevated in GBMs and associated with short survival in astrocytomas. On the other hand, the results obtained from survival analyses with FTH and FTL and the identified microglial expression suggest that iron-flux in gliomas may be dependent of different cell types and crosstalk between these cell types.

In conclusion, we found that TfR1 tumor cell expression increased with malignancy grade. No grade-dependent increase was found for the tumor cell expression of FTH and FTL, but the expression level of FTL by microglia was highest in GBMs. These findings suggest that iron-related proteins, primarily TfR1 and FTL, may be associated with tumor aggressiveness. The prognostic values of TfR1, FTH, and FTL in the individual astrocytoma grades were limited. However, high microglial/macrophage expression of FTL, but not FTH, correlated with shorter survival in all astrocytomas suggesting that development of future therapeutic strategies compromising iron flux should take tumors cells as well as microglia/macrophages into account.

## Supporting information

S1 Fig Association between iron-related mRNA levels and overall survival in the different GBM subtypes. In the classical GBM subtype, no significant association between overall survival and TfR1 **(A)**, FTH **(B)**, or FTL **(C)** was found. In the mesenchymal subtype, high levels of TfR1 correlated with poor prognosis **(D)**, while no significant correlation was found between overall survival and FTH **(E)** or FTL **(F)**. In neural GBMs, TfR1 **(G)**, FTH **(H)**, and FTL **(I)** did not correlate with survival. In proneural GBMs, high TfR1 was significantly associated with shorter overall survival **(J)**. Similar tendency was observed for FTH **(K)**, while no correlation existed between overall survival and FTL mRNA levels **(L)**.
*Abbreviations*: FTH ferritin heavy chain, FTL ferritin light chain, GBM glioblastoma, TfR1 transferrin receptor-1.
(TIF)

S1 File Clinicopathological data on the patients investigated in the current study. (XLSX)

S2 File Datasets generated from the Cancer Genome Atlas (TCGA). (XLSX)
