# Supplementary figures and images for: Transferrin receptor-1 and ferritin heavy and light chains in astrocytic brain tumors: Expression and prognostic value
Source: PLoS One. 2017 Aug 24;12(8):e0182954. doi: 10.1371/journal.pone.0182954 (PMC5570299; doi:10.1371/journal.pone.0182954)

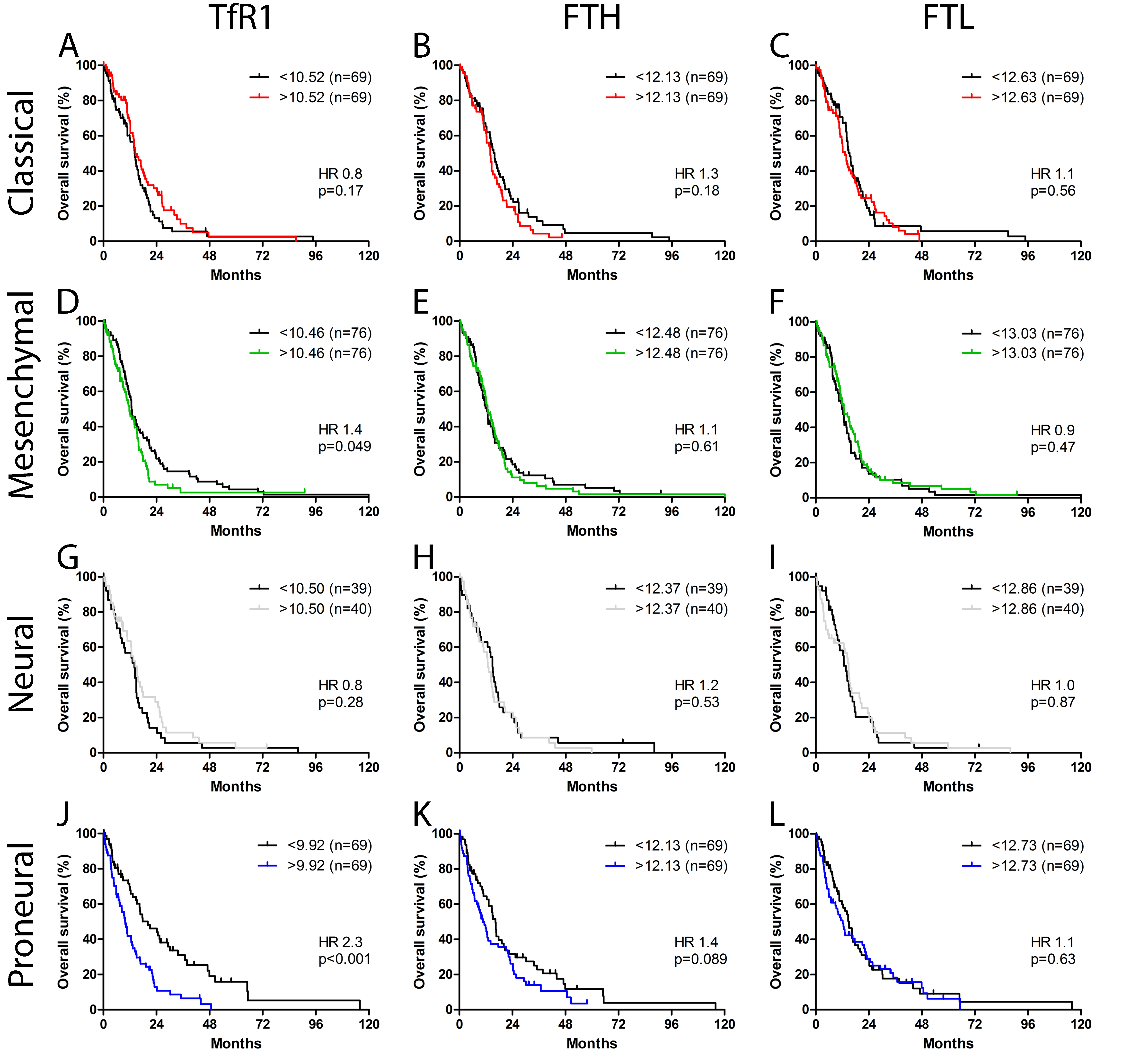

Supplement: S1 Fig — In the classical GBM subtype, no significant association between overall survival and TfR1 (A), FTH (B), or FTL (C) was found. In the mesenchymal subtype, high levels of TfR1 correlated with poor prognosis (D), while no significant correlation was found between overall survival and FTH (E) or FTL (F). In neural GBMs, TfR1 (G), FTH (H), and FTL (I) did not correlate with survival. In proneural GBMs, high TfR1 was significantly associated with shorter overall survival (J). Similar tendency was observed for FTH (K), while no correlation existed between overall survival and FTL mRNA levels (L). Abbreviations: FTH ferritin heavy chain, FTL ferritin light chain, GBM glioblastoma, TfR1 transferrin receptor-1. (TIF) [file pone.0182954.s001.tif]
